# Finding genes that influence quantitative traits with tree-based clustering

**DOI:** 10.1186/1753-6561-5-S9-S98

**Published:** 2011-11-29

**Authors:** Ian J Wilson, Richard AJ Howey, Darren T Houniet, Mauro Santibanez-Koref

**Affiliations:** 1 Institute of Genetic Medicine, Newcastle University, Newcastle NE3 1NB, UK

## Abstract

We present a new statistical method to identify genes in which one or more variants influence quantitative traits. We use the Genetic Analysis Workshop 17 (GAW17) data set of unrelated individuals as a test of the method on the raw GAW17 phenotypes and on residuals after fitting linear models to individual-based covariates. By performing appropriate randomization tests, we found many significant results for a proportion of the genes that contain variants that directly contribute to disease but that have an increased type I error for analyses of raw phenotypes. Power calculations show that our methods have the ability to reliably identify a subset of the loci contributing to disease. When we applied our method to derived phenotypes, we removed many false positives, giving appropriate type I error rates at little cost to power. The correlation between genome-wide heterozygosity and the value of the trait Q1 appears to drive much of the type I error in this data set.

## Background

Multilocus approaches to associations between variants and traits are likely to be advantageous when rare single-nucleotide polymorphisms (SNPs), which have an undetectable effect on a trait when considered singly, can explain a large proportion of the genetic variance at a locus when they are taken together [1-3]. Investigators have taken a gene-centric approach to association testing using, for example, entropy [4], weighted sums [2,3], and distance measures [5] to summarize information across different sites. Our approach uses data-driven tree-based clustering to combine genotypes across multiple loci. The tree structure makes our algorithm an efficient way to search through SNPs that best explain the difference in quantitative trait values. Our tree construction method ensures that genotypes that differ by a mutation at a single locus always cluster on the tree and gives an easily interpretable visualization of the SNPs at a locus that affects the trait.

Sevon et al. [6] developed a method, TreeDT, that uses lexical sorting of haplotypes to produce a tree-based test of association. We use the idea of lexical trees but extend it by using multilocus genotypes and by working with quantitative traits rather than case-control status. The method can be used on haplotypes, but using multilocus genotypes is a natural extension when we are interested in the effects of rare variants, because these variants are unlikely to be present in two copies and phasing of such variants is much more uncertain [7]. We develop this method to work with rare variants by using recoded multilocus genotypes rather than haplotypes and by extending the statistics used to look for associations between quantitative traits and the tree structure. Every node on the tree represents a multilocus genotype that appears one or more times in the population. Shorter multilocus genotypes are situated at internal nodes of the tree. This method provides a pictorial summary of the information contained in a region at different genes along the chromosome. The methods presented here are implemented both as a stand-alone program and as an R library [8].

## Methods

### Data preparation

For these analyses we use the unrelated individuals genotype data from Genetic Analysis Workshop 17 (GAW17); these data consist of 697 individuals genotyped at 22,487 sites. The data generation is detailed by Almasy et al. [9]. For tree analyses, we recode these genotype data as binary multilocus genotypes (BMGs) by coding homozygotes for the common allele as 0 and all other genotypes as 1, so that the BMG of an individual at a gene is a vector of 0’s and 1’s. This recoding is illustrated with example data in Table 1. We perform two sets of analyses: all preliminary work is done on the first phenotype data set, and subsequent power calculations are performed on all 200 data sets.

**Table 1:** Genotype frequencies for example data set

Multilocus genotype			
		
Allele count	Code	Control counts	Case counts
0-0-0-0	0-0-0-0	27	18
0-0-0-1	0-0-0-1	7	8
0-0-1-0	0-0-1-0	4	13
0-0-2-0			
0-0-1-1	0-0-1-1	0	6
0-0-1-2			
0-0-2-1			
0-0-2-2			
0-1-0-0	0-1-0-0	9	4
0-2-0-0			
1-0-0-0	1-0-0-0	4	0

The example data set is shown in Figure 2.

### Principal components and individual variation

**Figure 1:**
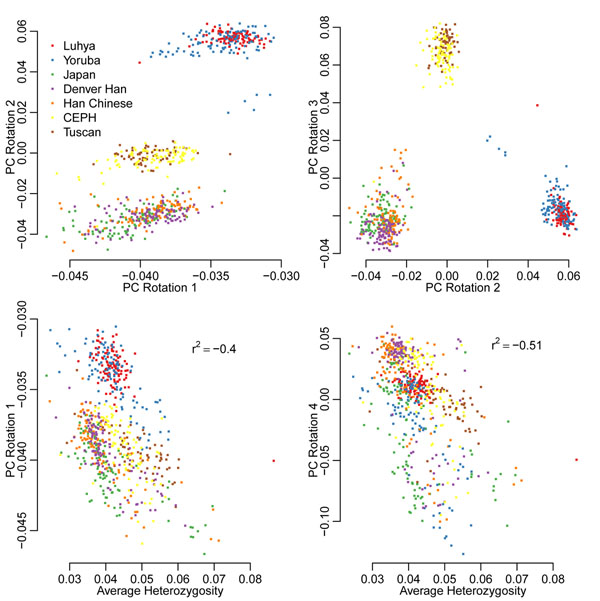
**Scatterplots of principal component loadings and summary statistics**. Heterozygosity is calculated by averaging over all sites in an individual. Values in the bottom two plots are Pearson correlation coefficients.

Loadings from principal components analysis (PCA) were calculated for allele counts for all genotype data using standard R functions. Plots of loadings are shown in Figure 1. The first principal component (PC), explaining 35% of the variance, does not resemble typical PCA results because it does not produce a separation into the three main population groupings that is seen in other studies with comparable samples [10], whereas rotations 2 and 3 effectively separate the individuals into three groups. Rotations 1 and 4 are more closely related to the overall variation seen in a sample (as measured by average heterozygosity) than to differences between populations, as seen for SNP array data [10].

### Derived phenotype data

To incorporate suspected relations between Q1, Q2, Q4, and the other explanatory variables (Age, Smoke, Sex, and PC loadings), we calculate two additional derived phenotypes for each of Q1, Q2, and Q4. We construct the first residual phenotype by fitting the linear model:(1)

where *ε* ~ *N*(0, *σ*^2^), using backwards stepwise selection on the explanatory variables and using the Bayesian information criterion [11] to decide which variables are retained. After model fitting, we use the standardized residuals as phenotypes. We calculate a second set of derived phenotypes in the same way but with the initial model also containing the first six variable PC loadings, which we label PC1, … , PC6.. All of this model selection is done on phenotype data set 1. We then fit the selected models individually to each of the 200 replicate data sets and use their standardized residuals as phenotype data. All calculations are performed using standard R functions.

### Building trees from genetic data

**Figure 2:**
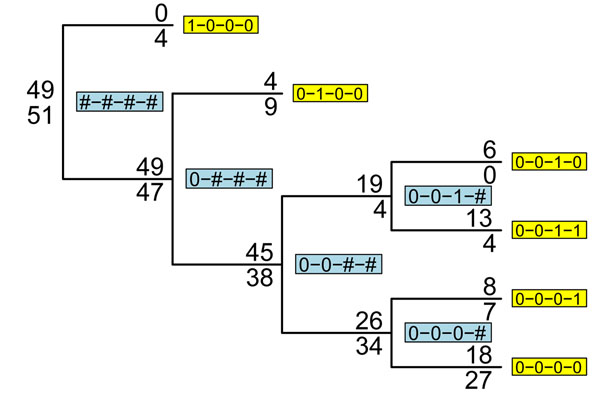
**Example of multilocus genotype tree.** This tree is constructed by considering sites left to right along the binary multilocus genotype (BMG). The root of the tree contains all 100 individuals. As we consider successive SNPs, all nodes containing both 0 and 1 individuals at the SNP are split, with individuals carrying a 1 put on the top branch and those carrying a 0 on the lower branch. The leaves of the tree (yellow background) carry full-length BMGs, and internal nodes (blue) carry partial BMGs, with sites that can carry 0 or 1 labeled with a hash (#). Case and control counts are given by numbers above and below the node label, respectively.

Consider a set of BMGs for a gene (here we could also use haplotypes if we had accurate phasing) as strings of 0’s and 1’s. Put all the individuals at the root of a tree. Now consider the variant positions in that gene one position at a time from left to right or using some other ordering. For the first position, all those samples with a 0 at the position are put on the left branch of the root, and all those with a 1 are put on the right branch. The two leaves of this tree now contain BMGs of length one 1. Now step through all the other variable positions for each leaf. If there is any variation at the current position among the individuals at a leaf, the leaf is split in two, with all individuals with a 0 on the left branch and all individuals with a 1 on the right branch. Repeat for all the variable sites at the gene. After *k* sites the leaves contain BMGs of length *k*. This procedure is illustrated in Figure 2. The multilocus genotypes that map to multilocus genotype codes for our example data are shown in Table 1.

### Test statistics

Obtaining a tree test statistic is a two-stage process. First, we require values of partial test statistics defined on the leaves and internal nodes of our tree. A variety of test statistics are available, but we are restricted to those that depend only on values of a trait at a node and its descendants. Using information at ancestral nodes or on nodes on other branches of the tree (such as other individuals from the same population) is not possible within this framework. We use the term *disjoint* here for a set of nodes in which none of the nodes is the ancestor of another. For further details see Sevon et al. [6].

**Figure 3:**
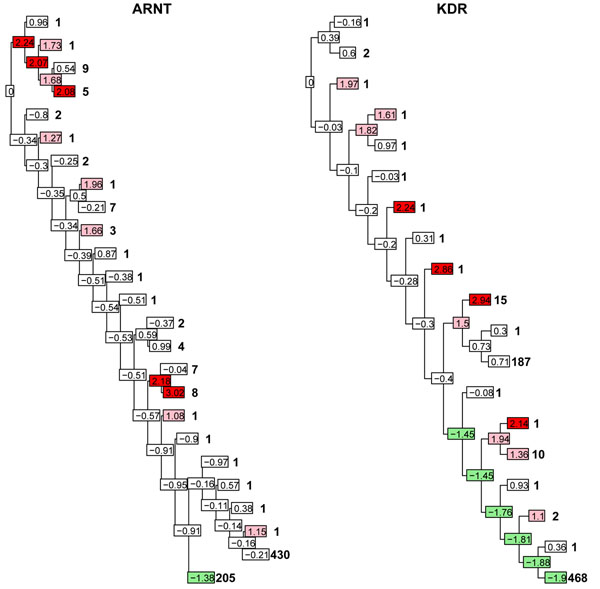
**Genotype trees underlying two disease positions**. Binary multilocus genotype trees underlying genes affecting phenotype Q1 from data set 1. *ARNT* is not significantly associated with Q1 using QTLTree, whereas *KDR* is associated. Numbers in boxes are values of the *z* statistic at the node; counts of individuals at the leaf are to the right. Counts for internal nodes can be calculated by looking at descendants. Colored boxes indicate evidence for association with phenotype within the tree: red, *z* > 2.0; pink, 1 ≤ *z* ≤ 2; and green, −2 ≤ *z* ≤ −1. Uncoloured boxes show no evidence of association with phenotype.

Although many partial test statistics are possible, we take a simple one, the *z* score:(2)

where *x_ij_*, *j* = 1, …, *n_i_*, are the values of the trait at node *i* and  and *s* are the sample mean and standard deviation, respectively, of the trait over all individuals. Two trees with the respective *z* scores at the nodes are shown in Figure 3.

The QTLTree test statistic over the whole tree, *S_k_*, is defined as the maximum value of:(3)

where the summation is over *m* disjoint nodes where *m* ≤ *k*, and *f* is some function. For our analyses, we take:(4)

that is, , but other approaches are possible and implemented in our program. As a side effect of the calculation, we obtain intermediate values for *S_j_*, *j* = 1, …, *k* − 1. Typically for our tests, we take *k* = 10.

### Significance testing

The null distribution of *S_k_* is not available. Because the GAW17 data were sampled from different populations, a straightforward randomization was not appropriate and individuals were randomized within populations; that is, the traits were randomly relabeled within each population so that the mean and standard deviation of the traits stayed the same within populations across replicate permutations. The seven different populations used were Luhya, Yoruba, Japanese, Denver Chinese, Han Chinese, CEPH (European-descended residents of Utah), and Tuscan.

We calculate statistical significance with 10^6^ randomized replicates per gene for the phenotype data set. We perform power calculations over multiple data sets using 10^5^ replicate simulations over all 200 data sets. One hundred thousand replicate permutations for all 3,205 genes of a single data set typically takes about 60 minutes, and these calculations are easy to perform in parallel, making them feasible for whole-genome data. The R package QTLTree [8] is available from IJW’s website (http://www.staff.ncl.ac.uk/i.j.wilson).

## Results

### Model choice derived phenotypes

The backwards model selection results in three additional derived phenotypes: Q1A, the residuals after fitting Age and Smoke; Q1B, the residuals after fitting Age + Smoke + PC1 + PC4; and Q4A, the standardized residuals after fitting a linear model with predictors Age + Sex + Smoke to Q4. No nonconstant terms were kept in models for Q2, and no extra PC terms were kept for Q4.

### Analysis of data set 1

**Figure 4:**
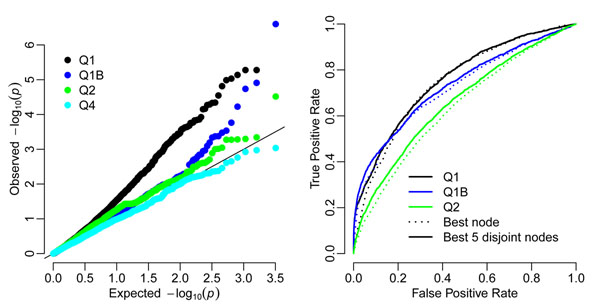
**Power calculations**. The left-hand plot gives a quantile-quantile plot for *p*-values from the analysis of data set 1. The solid line is the expected result. In the right-hand panel true-positive proportions are plotted against false-positives for varying significance level. This summarizes the results over all 200 replicate data sets, with solid lines denoting test statistics from five disjoint nodes (*S*_5_) and dotted lines denoting the maximum node value (*S*_1_).

Table 2 gives the genes with the highest significance levels for analyses of the three Q1-related traits and Q2. Although for the uncorrected trait the gene with rank 1 is true, the rest of the top 10 ranked genes are all unrelated to the trait and are significant after Bonferroni correction (*p* < 5 × 10^−5^). No improvement is seen after correcting for Age and Smoke. Only the derived trait Q1B behaves well. For Q2 there are no false positives after strict Bonferroni correction. Quantile-quantile plots of *p*-values from QTLTree tests of Q1, Q2, Q1B, and Q4 are shown in Figure 4. The *p*-values of phenotype Q4 follow their expectations well, whereas those for Q1 approach acceptability only when using phenotype Q1B, corrected using PC loadings. Results from phenotype Q2 show some deviation from the expected at low *p*-values, but these do not seem to be due to true associations from results in Table 2.

**Table 2:** Summary results for data set 1

Q1							Q2		
Gene	Q1 rank	Q1A rank	Q1B rank	*p*-value for Q1	*p*-value for Q1A	*p*-value for Q1B	Gene	Q2 rank	*p*-value for Q2

FLT1*	1	1	1	0.0000	0.0000	0.0000	*LRRC18*	1	0.000441
*ZNF91*	2	5	6	0.0000	0.0000	0.0002	*WDFY4*	2	0.000441
*FLJ22662*	3	22	900	0.0000	0.0000	0.2604	*SPAG8*	3	0.000453
*ZNF454*	4	3	27	0.0000	0.0000	0.0044	RARB*	24	0.004243
*KRT3*	5	12	134	0.0000	0.0000	0.0285	GCKR*	28	0.005489
*MAP2K6*	6	16	834	0.0000	0.0000	0.2360	VNN1*	42	0.00835
*ZNF568*	7	4	118	0.0000	0.0000	0.0244	BCHE*	358	0.063238
*BRCA1*	8	15	58	0.0000	0.0000	0.0115	SIRT1*	382	0.068709
*RGPD8*	9	7	140	0.0000	0.0000	0.0301	INSIG1*	391	0.07131
KDR*	14	2	15	0.0001	0.0000	0.0018	VNN3*	639	0.128922
VEGFC*	15	238	26	0.0001	0.0090	0.0044	LPL*	1,443	0.382003
VEGFA*	157	197	282	0.0086	0.0062	0.0717	PLAT*	1,833	0.514235
*ELAVL4*	312	729	1,248	0.0281	0.0983	0.3655	VWF*	2,071	0.59704
ARNT*	653	1,086	983	0.1025	0.2037	0.2872	PDGFD*	2,426	0.716842
FLT4*	1,352	788	1,757	0.3187	0.1173	0.5233	VLDLR*	2,691	0.811708
HIF1A*	1,563	998	1,660	0.3930	0.1814	0.4937	SREBF1*	2,891	0.883901

All *p*-values based on 10^6^ randomizations. Ranks are the rank of the genes when ordered by *p*-value. Genes with asterisks indicate true associations with trait. Genes were chosen on the basis of rank for Q1 analyses or of being true.

### Power calculations

Power calculation results are summarized in Figure 4. There is some power to detect true associations at genes influencing traits Q1 and Q2, but not for all genes. Using the derived trait Q1B, which incorporates PC loadings, increases the true-positive rate at low false-positive rates. Using five disjoint test statistics increases the power over using a single statistic. Further test statistics did not further increase power (results not shown). Table 3 shows that there is a tendency for false positives to be found at the same genes over all replicate simulations, even for the derived traits. This table also informs us that although phenotype Q4 is well behaved for data set 1, across all data sets there is a tendency for false-positive results to be seen in the same genes. Using the residual derived phenotype Q4A corrected this problem (results not shown).

**Table 3:** Summaries of repeatable significant results over 200 data sets

Q1		Q1A		Q1B		Q2		Q4	
Gene	*n* ≤ 0.01	Gene	*n*	Gene	*n*	Gene	*n*	Gene	*n*

***FLT1***	**200**	***FLT1***	**200**	***FLT1***	**191**	***VNN1***	**67**	*BUD13*	170
*FLJ22662*	192	***KDR***	**200**	***KDR***	**135**	*OR5B2*	48	*SLC22A1*	154
*ZNF713*	188	*TERT*	197	*MAP2K7*	135	*PTGIS*	39	*SEPT1*	129
***KDR***	**187**	*ZNF713*	196	*FOXO3*	133	*ZNF568*	38	*CYP3A43*	116
*FOXO3*	170	*C1ORF147*	194	*HSZFP36*	94	*METTL2B*	32	*TOB2*	115
*KRT3*	170	*FLJ22662*	194	*EPHB1*	90	***GCKR***	**32**	*NF2*	85
*KRT75*	164	*OR6C4*	192	*LRP4*	88	*MUC19*	31	*NUP188*	83
*GRK1*	162	*PRKCH*	192	*RBM6*	86	*UNC45B*	31	*OR10T2*	68
*E2F2*	157	*ALX4*	190	*GRIA4*	79	*ZNF518B*	31	*C16ORF55*	67
*CETP*	155	*E2F2*	189	*FNDC3A*	78	***VNN3***	**29**	*ICAM4*	65
*TERT*	155	*ETV6*	189	*HIST2H2BE*	73	***SIRT1***	**25**	*LOC1001316*	65
*MAP2K6*	154	*KRT75*	189	*HAS3*	72	***BCHE***	**22**	*MYCBP*	65
***VEGFC***	**45**	***VEGFC***	**65**	***VEGFC***	**65**	***SREBF1***	**16**	*WBP1*	65
***ARNT***	**38**	***HIF1A***	**51**	***VEGFA***	**19**	***LPL***	**14**	*MUC3A*	61
***VEGFA***	**32**	***FLT4***	**51**	***ARNT***	**18**	***RARB***	**11**	*CNGA3*	58
***FLT4***	**51**	***VEGFA***	**32**	***FLT4***	**7**	***PDGFD***	**8**	*GTSE1*	58
***ELAVL4***	**15**	***ARNT***	**37**	***HIF1A***	**7**	***VLDLR***	**7**	*MMP27*	58
***HIF1A***	**12**	***ELAVL4***	**12**	***HIF3A***	**2**	***VWF***	**3**	*PIK3R2*	58
***HIF3A***	**3**	***HIF3A***	**2**	***ELAVL4***	**2**	***INSIG1***	**1**	*TAAR5*	56

All significant results are based on a noncorrected significance level of 0.01. Numbers are the number of replicates where a gene was found to be significant (out of 200). Columns show the top results and those from true genes (bold). The maximum number of significant tests for Q4A was 8. All significance tests are based on 10^5^ permutations.

## Discussion and conclusions

The methods in QTLTree described here are a quick way to collapse the information contained in genotypes within a gene into a form that allows quick calculation of an optimum set of SNPs or combination of SNPs. Within the R environment, the method can also be used to interactively explore the sets of SNPs that may be affecting a quantitative trait. The methods seem to be able to detect an appreciable proportion of genes underlying variation in phenotypes, although the large number of detected loci that do not contribute to variation using the raw trait data is worrying, because within-population randomization should account for any simple differences between populations. Because different levels of structure exist within the data and because gene-environment-region interactions are possible (e.g., the age structure differs between populations, and the values of the Q1 and Q4 phenotypes depend on age), we attempted a further level of correction. Using derived phenotypes after regressing on correlated phenotypes and PC loadings improved the type I error rates while not reducing the power for realistic false-positive rates.

Plots of PC loadings produced some unusual results that looked different from those from SNP arrays [8]. These are also seen in the correlations of Table 4, where PC loadings 1, 2, and 4 are significantly correlated with phenotypes Q1 and Q4. Because, from the answers to the GAW17 simulation, the sequenced genes have no direct effect on phenotype Q4, the association of Q4 with PC loading 2 is most likely through both being associated with age. The correlation disappears when we take the residual after correcting for age, sex, and smoking status. Associations with the first loading, which explains more than 30% of the variation in the sample, are more difficult to explain because the first loading is correlated with the average heterozygosity (Figure 1; Table 4). Although this may reflect an underlying variation in heterozygosity between people, it seems more likely that it reflects differences in coverage in sequencing samples, because samples with higher coverage tend to have more variants called.

**Table 4:** Correlations between population statistics and phenotypic traits

Trait	Average heterozygosity	Rotation 1	Rotation 2	Rotation 3	Rotation 4
Age	0.02	**0.14**	**0.16**	0.07	0.06
Smoke	−0.01	−0.04	−0.05	0.04	0.01
Q1	**0.16**	**−0.15**	−0.07	0.05	**−0.19**
Q2	0.05	−0.01	0.01	−0.04	−0.05
Q4	−0.06	−0.09	**−0.12**	−0.07	−0.03
Q1A	**0.17**	**−0.21**	−0.12	0.02	**−0.24**
Q1B	0.02	0.00	−0.01	−0.01	0.00
Q4A	−0.10	0.06	0.02	−0.02	0.06
Affected	**0.14**	0.02	0.09	0.07	−0.05

Correlations are Pearson product moment statistics. Results significant at the 0.1% level are indicated in bold. Definitions of residual phenotypes Q1A, Q1B, and Q4A are given in the text.

The strong false-positive signals with the raw data lead us to ask, can the difference in the number of variable sites between individuals explain the inflated errors in Q1? To test this for phenotypes Q1 and Q2, we created data sets with just the SNPs that affected disease and all non-disease-causing SNPs. The correlation across individuals between average heterozygosity for SNPs affecting Q1 and average heterozygosity for SNPs not affecting Q1 is 0.22 (Pearson *r*^2^, *p* = 5.6 × 10^−9^), and the correlation for Q2 is *r*^2^ = 0.14 (*p* = 2 × 10^−4^). This may explain some of the false positives. Although such problems are unlikely to arise for real data, they emphasize the difficulties that may crop up in future studies using next-generation sequencing technologies if case and control subjects are not treated in the same way and if genotyping and variant calling are not performed blind to disease status.

## Competing interests

The authors declare that there are no competing interests.

## Authors’ contributions

IJW wrote the computer code, carried out statistical analyses and drafted the manuscript. RAJH and DTH assisted with statistical analyses and helped to draft the manuscript. MSK conceived the statistical methodology. All authors read and approved the final manuscript.
